# MeioBase: a comprehensive database for meiosis

**DOI:** 10.3389/fpls.2014.00728

**Published:** 2014-12-16

**Authors:** Hao Li, Fanrui Meng, Chunce Guo, Yingxiang Wang, Xiaojing Xie, Tiansheng Zhu, Shuigeng Zhou, Hong Ma, Hongyan Shan, Hongzhi Kong

**Affiliations:** ^1^State Key Laboratory of Systematic and Evolutionary Botany, Institute of Botany, Chinese Academy of SciencesBeijing, China; ^2^University of Chinese Academy of SciencesBeijing, China; ^3^State Key Laboratory of Genetic Engineering and Collaborative Innovation Center for Genetics and Development, Ministry of Education Key Laboratory of Biodiversity Sciences and Ecological Engineering, Institute of Plant Biology, Institute of Biodiversity Science, Center for Evolutionary Biology, School of Life Sciences, Fudan UniversityShanghai, China; ^4^Shanghai Key Lab of Intelligent Information Processing and School of Computer Science, Fudan UniversityShanghai, China; ^5^Institutes of Biomedical Sciences, Fudan UniversityShanghai, China

**Keywords:** meiosis, MeioBase, knowledge database, meiotic genes, protein-protein interaction, eukaryotes, sexual reproduction

## Abstract

Meiosis is a special type of cell division process necessary for the sexual reproduction of all eukaryotes. The ever expanding meiosis research calls for an effective and specialized database that is not readily available yet. To fill this gap, we have developed a knowledge database MeioBase (http://meiosis.ibcas.ac.cn), which is comprised of two core parts, *Resources* and *Tools*. In the *Resources* part, a wealth of meiosis data collected by curation and manual review from published literatures and biological databases are integrated and organized into various sections, such as *Cytology, Pathway, Species, Interaction*, and *Expression*. In the *Tools* part, some useful tools have been integrated into MeioBase, such as *Search, Download, Blast, Comparison, My Favorites, Submission*, and *Advice*. With a simplified and efficient web interface, users are able to search against the database with gene model IDs or keywords, and batch download the data for local investigation. We believe that MeioBase can greatly facilitate the researches related to meiosis.

## INTRODUCTION

Meiosis is a specialized cell division process essential for all sexually reproducing organisms. During meiosis, a single round of DNA replication is followed by two successive rounds of nuclear division, meiosis I and meiosis II. Meiosis I is unique and involves the segregation of homologous chromosomes (homologs), whereas meiosis II is similar to mitosis and results in the segregation of sister chromatids. The function of meiosis is to generate four haploid gametes, which are able to develop into germ cells. Fertilization of the germ cells, then, can restore the offspring to the chromosome number and complexity level of their parents (Zickler and Kleckner, 1998; Hamant et al., 2006). Meiosis not only ensures the stability of chromosome numbers between generations, but also provides genetic materials for biodiversity.

Studies of meiosis have been carried out extensively for over 100 years (Hamant et al., 2006). Chromosome behaviors in some species have been examined in detail by using cytological approaches (Orr-Weaver, 1995; Zickler and Kleckner, 1999; Ma, 2005; Birchler and Han, 2013). Over the last two decades, much efforts have been devoted to understanding the genetic basis and molecular mechanisms of meiosis in model species, such as nematode (*Caenorhabditis elegans*), budding yeast (*Saccharomyces cerevisiae*), *Arabidopsis* (*Arabidopsis thaliana*), rice (*Oryza sativa*), and maize (*Zea mays*). Genes regulating meiosis, especially those involved in homologous chromosome paring, synapsis, recombination and separation in prophase I, have also been cloned and characterized in terms of their functions (Hollingsworth et al., 1990; Sym et al., 1993; Keeney et al., 1997; Klimyuk and Jones, 1997; Yang et al., 1999; Li et al., 2004; Tang et al., 2014). Recent advances in transcriptomics, protein-protein interactions (PPIs), and phylogenetic analyses of genes and gene families related to meiosis have improved our understanding of this complex process dramatically (Kee and Keeney, 2002; Wang et al., 2004; Lin et al., 2006; Vignard et al., 2007; Chen et al., 2010; Tang et al., 2010; Yang et al., 2011; Dukowic-Schulze et al., 2014).

The ever expanding studies of meiosis and the data accumulated, which are scattered in tremendously diverse literatures and a few of large databases, such as NCBI, Ensemble, and TAIR, call for an integrative and encyclopedia-like platform for meiosis (Hamant et al., 2006; Handel and Schimenti, 2010; Luo et al., 2014). Recently, two databases related to reproductive development, GermOnline and Plant Male Reproduction Database (PMRD), have been established. GermOnline is a cross-species microarray expression database focusing on germline development, reproduction and cancer (Lardenois et al., 2010). PMRD is a comprehensive resource for browsing and retrieving knowledge about genes and mutants related to plant male reproduction (Cui et al., 2012). Notwithstanding, neither databases provide comprehensive information about meiosis, because data related to meiosis is heavily fragmented. Therefore, researchers are in great need of effective tools or databases to quickly obtain precise meiotic data from the exponentially increasing amount of information.

Here, by collecting and integrating all sorts of information related to meiosis, as well as including and developing powerful tools for search, comparison and analysis, we established a comprehensive and specialized database for meiosis, MeioBase. It will not only serve the meiosis research community, but also help any users who need an easy and efficient access to various kinds of information related to meiosis.

## DATABASE STRUCTURE AND WEB INTERFACE

**FIGURE 1:**
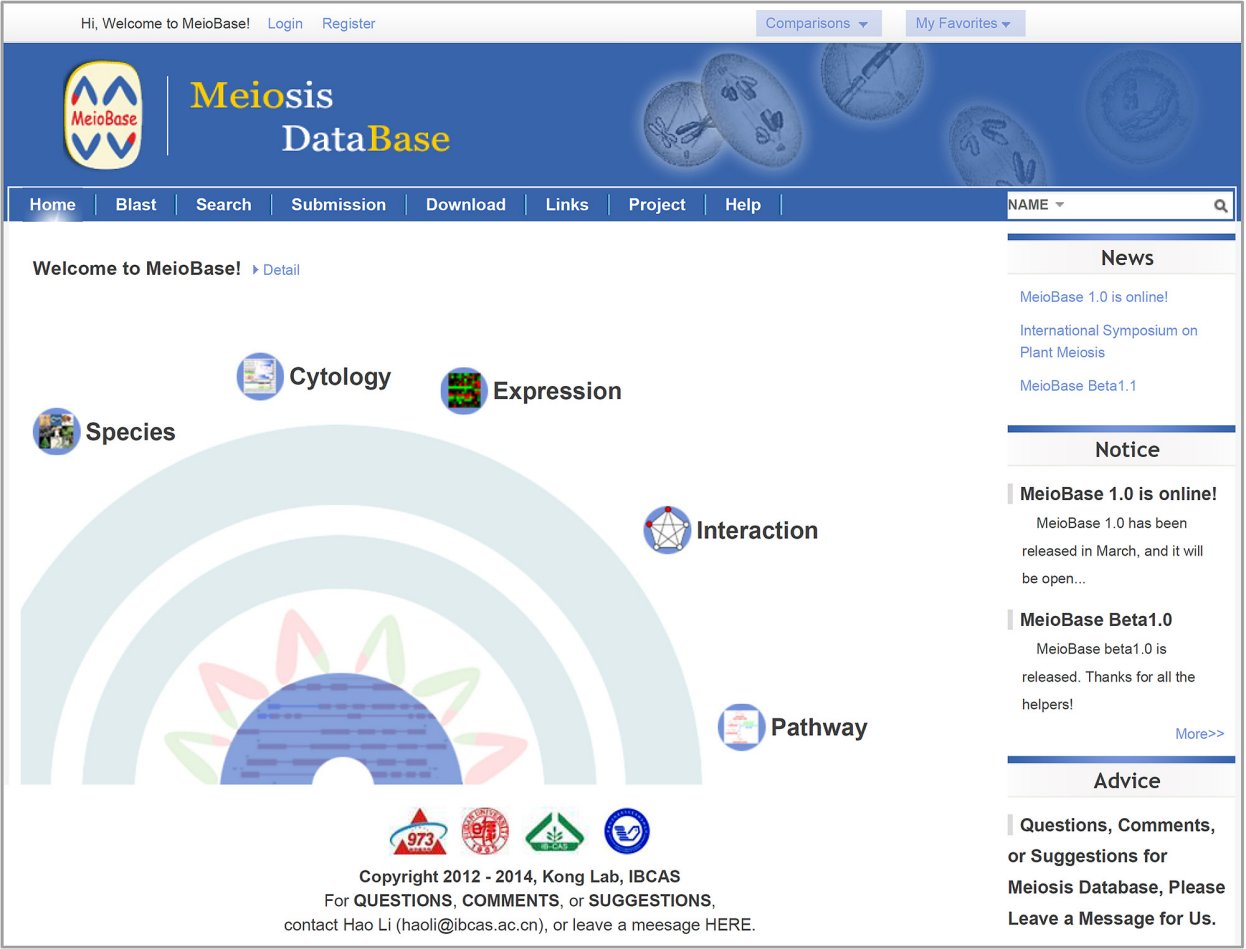
**Screenshot of MeioBase homepage**.

The database intends to provide all necessary resources and tools for meiosis-related researches (**Figure 1**). In the *Resources* part, information related to meiosis are categorized and integrated into five major sections (i.e., *Cytology*, *Pathway*, *Species*, *Interaction*, and *Expression*). In the *Tools* part, useful functions for searching, analyzing, uploading, and downloading data are included in seven sections (i.e., *Search*, *Download*, *Blast*, *Comparison*, *My Favorites*, *Submission*, and *Advice*). This structure can provide a centralized and user-friendly web portal for meiosis-related studies.

**FIGURE 2:**
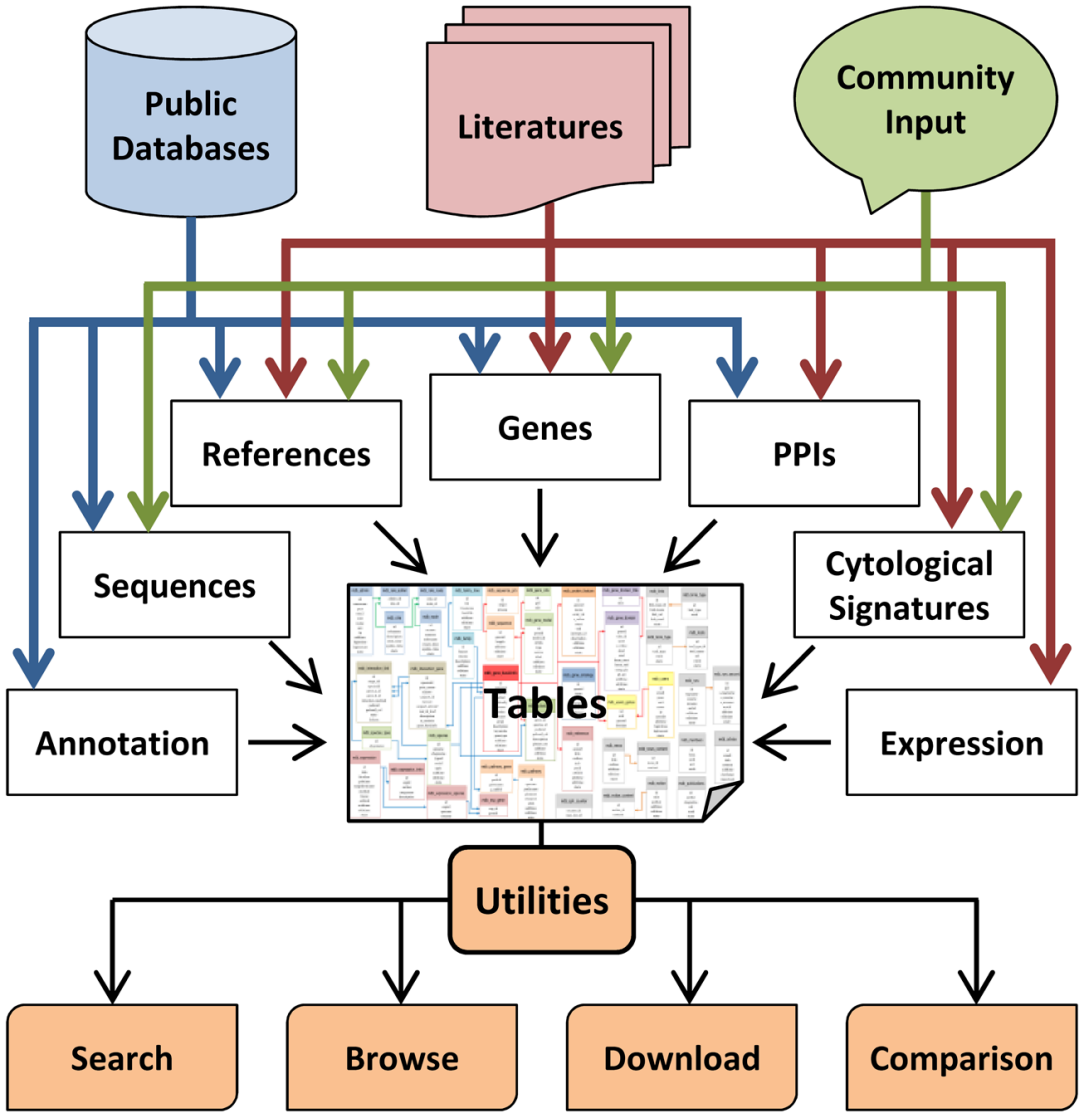
**The flowchart for construction of MeioBase.** MeioBase is a comprehensive database for browsing and retrieving knowledge about meiosis, meiotic genes, and related data. MeioBase brings together three main sources of knowledge: (1) basic information about genes from public databases; (2) detailed curation of meiosis-related studies from the literatures; (3) public contributions from research community and other users. All of the information is stored in relational database tables that could be accessed by the utilities of MeioBase through any web browser.

The website is built on a Linux, Apache, MySQL, and PHP (LAMP) stack. MySQL is used for storage, maintenance, and operation of the database, and 44 data sheets have been designed to form a network storing all the data (**Figure 2**). The front–end interface is implemented in PHP, which is a popular scripting language for dynamic web page. A well-defined and packaged JavaScript called jQuery is used to enhance the interface of the website and improve user experience. A navigation tool bar containing search box and links, such as *Home*, *Blast*, *Submission*, *Download*, and *Help*, are also included in each web page.

## RESOURCES

Cytological features of meiosis have been described in detail for many species, which laid a solid foundation for the study of the molecular basis of meiosis (Zickler and Kleckner, 1999; Ma, 2005). Here, in the *Cytology* section, we provide an overview of the cytological process of meiosis, with special emphasis on the chromosome behaviors at different stages. To help understand the conservation and variability of the meiotic process during evolution, cytological features in model species are summarized and compared. Important advances related to meiosis can also be retrieved through clicking the *Updated Advances* links from the overview page.

During meiotic prophase I, several critical events related to meiotic chromosome structure and interaction occur, including pairing, synapsis, recombination, and segregation. Consistent with this, genes regulating meiosis have also been grouped into pathways or networks, each of which corresponds to one of the meiotic events (Gerton and Hawley, 2005; Chang et al., 2011). For this reason, we have established the *Pathway* section, in which genes with similar or related functions, as well as the complex regulatory relationships between them, are visualized by diagrams.

**FIGURE 3:**
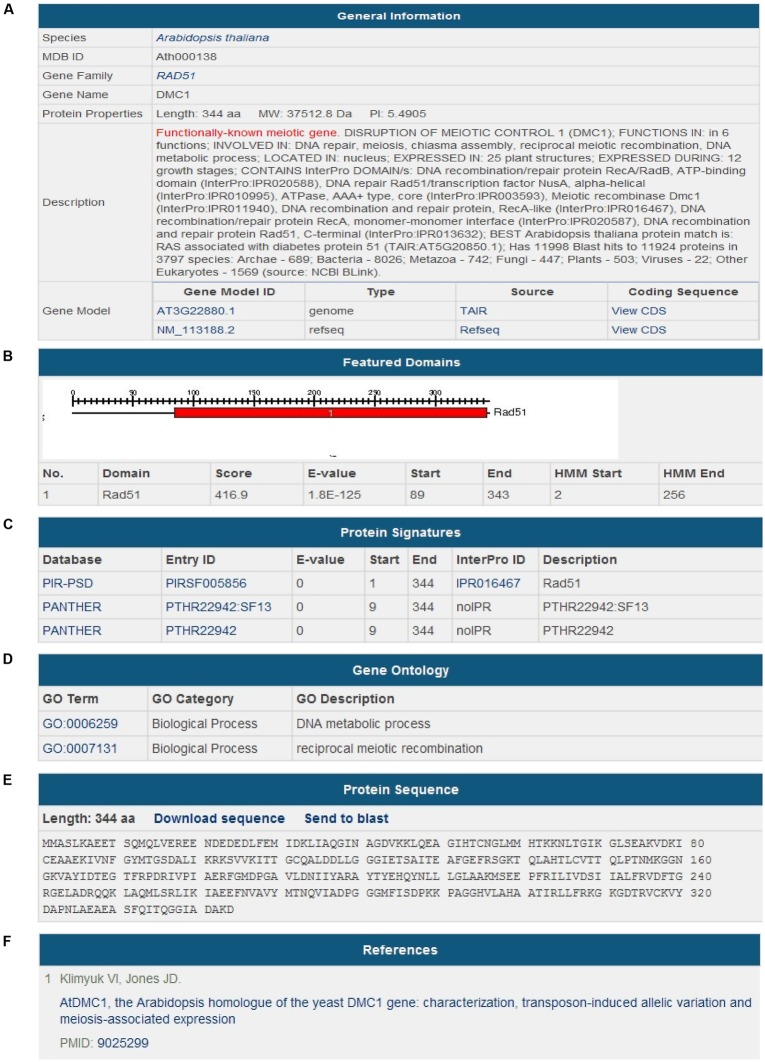
**Screenshot of gene information.** The *Gene Detailed Information* page of a meiotic gene consists of six aspects.** (A)** *General Information*; ** (B)** *Featured Domains*;** (C)** *Protein Signatures*;** (D)** *Gene Ontology*;** (E)** *Protein Sequence*;** (F)** *References*.

During the last 20 years, many genes involved in meiosis have been discovered and functionally characterized. By literature mining, 483 meiotic genes have been collected (**Table 1**). In the *Species* section, users can find the genes of a certain species and go into the details. In the *Gene Detailed Information* page, we have integrated useful information from references, databases and web servers. Data are organized by different aspects, such as *General Information*, *Featured Domains*, *Protein Signatures*, *Gene Ontology*, *Protein Sequence*, and *References*. *General Information* contains species name, gene name, gene family name, a brief description, gene model ID, and the coding sequence (CDS) of the gene. *Featured Domains* provides protein domains predicted by Pfam. *Protein Signatures* and *Gene Ontology* provide more information of the functions of the proteins. *References* includes literatures with PMIDs in PubMed (**Figure 3**).

**Table 1:** Data status in the current MeioBase.

Species	Common name	Number of meiotic genes						PPIs
		Total	Initiation & Pairing	Synaptonemal complex	Chromosomal recombination	Chromosome segregation	Unclassified
*Arabidopsis thaliana*	*Arabidopsis*	88	17	8	37	11	15	445
*Oryza sativa*	Rice	32	8	4	18	2	0	NA
*Zea mays*	Maize	8	4	1	2	1	0	NA
*Caenorhabditis elegans*	Nematode	178	16	11	31	62	58	675
*Mus musculus*	Mouse	10	0	6	4	0	0	NA
*Saccharomyces cerevisiae*	Budding yeast	162	18	9	71	22	42	10081
*Schizosaccharomyces pombe*	Fission yeast	5	1	0	3	1	0	NA
Total		483	64	39	166	99	115	11201

*^1^The number of functionally characterized meiotic genes that have been integrated into MeioBase.*

*^2^The number of PPI data that have been integrated into MeioBase.*

We also include 11,201 pieces of PPI data of the collected meiotic genes of *Arabidopsis*, nematode, and budding yeast in MeioBase, and display them in the *Interaction* section (**Table 1**). The PPI data are retrieved from 283 literatures and 11 databases, such as BIND, BioGRID, DIP, IntAct, and STRING. Users can search for partners of the proteins of interest, for which corresponding sources and references are provided.

To provide more comprehensive information on meiosis, references related to gene expression patterns are collected and displayed in the *Expression* section. Until now, important references of various species, such as *Arabidopsis*, rice, maize, petunia, wheat, mouse, rat, budding yeast, and fission yeast, have been listed in this section and more expression data of meiosis are being collected.

In addition to the aforementioned five sections, MeioBase provides many other resources in the *Links* section, such as the commonly used databases, powerful web servers for molecular and genomic analysis, and experimental protocols for meiosis research, etc. An introduction to our “Plant Meiosis Project” can be found in the *Project* section. Important progresses and events on meiosis research are available in the *News* section. Release notes of MeioBase are announced in the *Notice* section. To provide an overview of our database, we also include a detailed introduction in the *Help* section.

## TOOLS

MeioBase provides various ways to retrieve the data that users are interested in. By using the search box, users can search genes by gene names, gene model IDs or MeioBase IDs. In the *Search* section, users can search genes not only by inputting their model IDs but also by providing keywords describing them. PPI data can also be searched with keywords. All data can be downloaded in the *Download* section, which links to the FTP site.

Blast search against all the data in MeioBase is provided to facilitate users of finding similar sequence of a given sequence. In the *Comparison* section, users can add any two genes in the gene list and compare them. Users can also add any ten genes into the *My Favorites* section for fast checking afterward.

Moreover, we have integrated sections specifically for contributing meiosis data or suggestions to MeioBase. In the *Submission* section, users can first download and fill in a customized excel file as directions with meiotic genes, PPI data, and other data not yet included in the database, and then upload it to the database. After checking the uploaded data, we will add the qualified ones into MeioBase timely. In *Advice* section in homepage and at the foot of every page, users could ask any questions or give us comments or suggestions about this database. We will appreciate every user for improving MeioBase and reply as soon as possible.

## FUTURE PLANS

MeioBase is the first web database providing comprehensive information on meiosis. It is only a start of establishing a large and well-known database on meiosis. We will reiterate the process of database structure and user interface development to enhance the data content and functionality. The major data content enhancement will come from elaboration of the gene annotation and incorporation of more meiotic genes in other species, various expression data from references and other databases, PPI networks of different species, and other vital pathways during meiosis.

## Conflict of Interest Statement

The authors declare that the research was conducted in the absence of any commercial or financial relationships that could be construed as a potential conflict of interest.
